# CNEwrap: a scalable toolkit with a novel algorithm for large-scale genome-wide accelerated conserved non-coding elements detection

**DOI:** 10.1093/nar/gkag709

**Published:** 2026-07-20

**Authors:** Ruihan Li, Wei Wu, Chaochao Yan, Jia-Tang Li

**Affiliations:** China-Croatia Belt and Road Joint Laboratory on Biodiversity and Ecosystem Services and National Engineering Research Center for Natural Medicines, Chengdu Institute of Biology, Chinese Academy of Sciences, 610213, Chengdu, China; Mountain Ecological Restoration and Biodiversity Conservation Key Laboratory of Sichuan Province and Key Laboratory of National Forestry and Grassland Administration on Biodiversity Conservation on the Qinghai-Xizang Plateau, Chengdu Institute of Biology, Chinese Academy of Sciences, 610213, Chengdu, China; China-Croatia Belt and Road Joint Laboratory on Biodiversity and Ecosystem Services and National Engineering Research Center for Natural Medicines, Chengdu Institute of Biology, Chinese Academy of Sciences, 610213, Chengdu, China; Mountain Ecological Restoration and Biodiversity Conservation Key Laboratory of Sichuan Province and Key Laboratory of National Forestry and Grassland Administration on Biodiversity Conservation on the Qinghai-Xizang Plateau, Chengdu Institute of Biology, Chinese Academy of Sciences, 610213, Chengdu, China; China-Croatia Belt and Road Joint Laboratory on Biodiversity and Ecosystem Services and National Engineering Research Center for Natural Medicines, Chengdu Institute of Biology, Chinese Academy of Sciences, 610213, Chengdu, China; Mountain Ecological Restoration and Biodiversity Conservation Key Laboratory of Sichuan Province and Key Laboratory of National Forestry and Grassland Administration on Biodiversity Conservation on the Qinghai-Xizang Plateau, Chengdu Institute of Biology, Chinese Academy of Sciences, 610213, Chengdu, China; University of Chinese Academy of Sciences, 101408, Beijing, China; China-Croatia Belt and Road Joint Laboratory on Biodiversity and Ecosystem Services and National Engineering Research Center for Natural Medicines, Chengdu Institute of Biology, Chinese Academy of Sciences, 610213, Chengdu, China; Mountain Ecological Restoration and Biodiversity Conservation Key Laboratory of Sichuan Province and Key Laboratory of National Forestry and Grassland Administration on Biodiversity Conservation on the Qinghai-Xizang Plateau, Chengdu Institute of Biology, Chinese Academy of Sciences, 610213, Chengdu, China; University of Chinese Academy of Sciences, 101408, Beijing, China; Southeast Asia Biodiversity Research Institute, Chinese Academy of Sciences, 05282, Nay Pyi Taw, Myanmar

## Abstract

Conserved non-coding elements (CNEs) are fundamental components of gene regulatory networks in eukaryotes, yet their reliable identification across large-scale genomes and systematic evaluation of their genetic variation remains technically challenging, limiting comprehensive insights into their functional roles. To address these challenges, CNEwrap (https://github.com/YanCCscu/CNEwrap) was developed as a streamlined and modular bioinformatics toolkit that integrates subprograms capable of performing diverse tasks ranging from whole-genome alignment to CNE scanning and accelerated evolution analysis. Designed for high-throughput, multi-species applications, CNEwrap enables efficient and accurate discovery of genome-wide CNEs and comparative analysis of their variation across diverse taxa. Specifically, we developed a novel algorithm, “EvoAcc,” designed for assessing accelerated evolution of specific species in different scenarios from CNE alignments. The EvoAcc algorithm integrates nucleotide variation frequencies and phylogenetic relationships to reconcile global conservation with clade-specific divergence, outperforming PhyloAcc, PhyloP, and ForwardGenomics in simulated datasets, particularly in scenarios involving two or three accelerated lineages. In validation analyses of functional genomic fragments across mammal species, EvoAcc performed comparably to existing algorithms in detecting human-specific accelerated segments while exhibiting superior sensitivity for InDel mutations and recovering specific signals missed by other algorithms. Case studies further confirm that CNEwrap is broadly applicable within diverse evolutionary lineages. Collectively, the CNEwrap pipeline establishes a scalable and integrative framework for uncovering CNEs and their evolutionary dynamics, while the incorporated EvoAcc algorithm complements existing methodologies, deepening insights into conserved regulatory architectures across eukaryotic evolution.

## Graphical Abstract

**Graphical Abstract:**
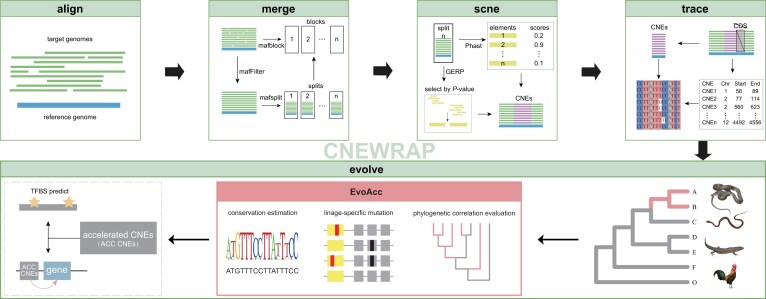

## Introduction

**Figure 1.**
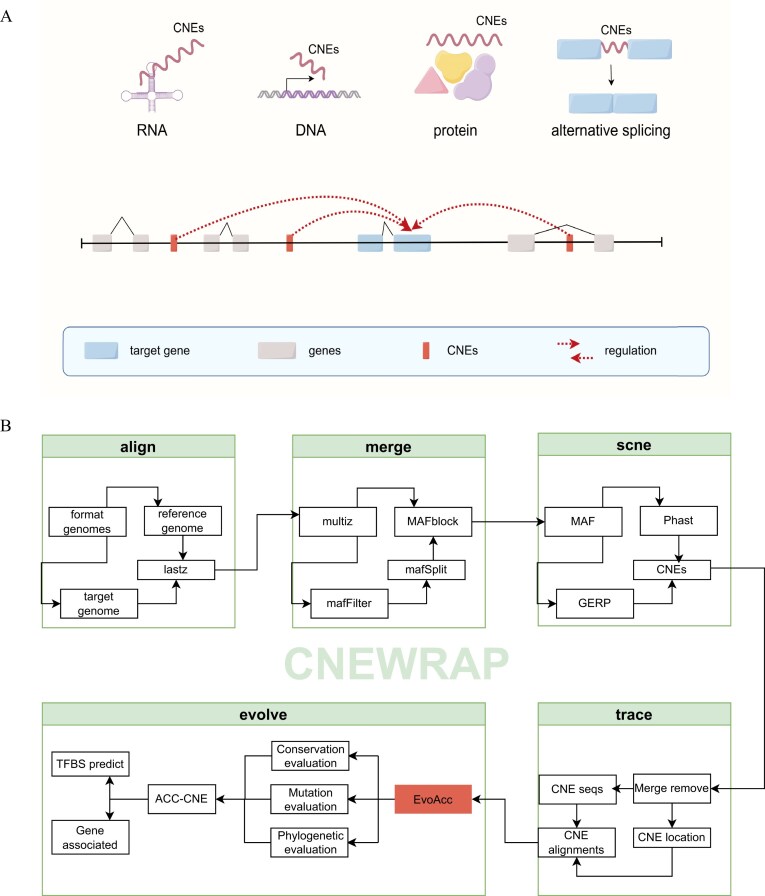
Functional roles of CNEs and the CNEwrap workflow. (**A**) CNEs interact with RNA, DNA, proteins, and intronic regions involved in alternative splicing. Illustration created using Figdraw (www.figdraw.com). (**B**) Overview of the five core subprograms in the CNEwrap pipeline. ACC-CNE indicates CNEs undergoing accelerated evolution.

Non-coding regions of the genome play a crucial role in regulating gene expression and shaping evolutionary trajectories across species. These regions exert their influence through various mechanisms, including enhancer activity and the recruitment of transcription factors to specific binding sites. Within this non-coding landscape, conserved non-coding elements (CNEs) represent a distinct class of sequences first identified through cross-species comparisons of mammalian and avian mRNAs [1–4]. Subsequent research has revealed the widespread conservation of these elements across a wide range of metazoan lineages [5, 6]. Remarkably, CNEs typically demonstrate higher sequence conservation than protein-coding exons and are consistently located in proximity to functional genes, serving as developmental enhancers [7]. CNEs may exert regulatory effects either through their transcription into non-coding RNAs (ncRNAs) that interact with DNA, RNA, or proteins or by residing within intronic regions where they can modulate alternative splicing events (Fig. 1A) [8]. The regulatory functions of ncRNAs are diverse and include transcriptional activation and repression, epigenetic regulation, mRNA splicing, translational inhibition, protein targeting or scaffolding, and RNA methylation [8, 9]. Functional disruption of CNEs through mutation has been shown to induce pronounced phenotypic consequences during vertebrate embryogenesis [10–13]. For example, avian lineage-specific CNEs regulate the expression of developmental genes such as *SIM1*, contributing to the formation of flight feathers [14]. Similarly, the M280 element has been identified as an essential enhancer driving limb development in mice [15]. Additionally, point mutations within the SHH ZRS enhancer have been causally linked to preaxial polydactyly in both humans and mice [12]. Collectively, the discovery and characterization of CNEs have substantially advanced mechanistic understanding of the molecular basis underlying the evolution of complex morphological traits.

Efforts to identify CNEs have consistently revealed their characteristic enrichment in AT-rich sequences, a feature first noted in comparative analyses of human and pufferfish genomes [16]. Multiple strategies have been developed for the classification of genomic elements, ranging from structural-based approaches [17] to those utilizing feature vector construction and rule-based classification frameworks [18]. CNEs exhibit sequence signatures that are both highly conserved and distinct from protein-coding exons, allowing them to be differentiated based on their evolutionary and compositional properties. The identification of CNEs primarily depends on robust whole-genome alignment tools, which provide the resolution necessary to detect conserved sequences across divergent lineages, such as LASTZ [19], Cactus [20], and MULTIZ [21]. Following alignment, conserved elements can be detected using phylogenetic and evolutionary constraint-based tools, including Genomic Evolutionary Rate Profiling (GERP) [22] and phastCons [23].

In recent years, accelerated evolution of CNEs has emerged as a critical area of investigation in studies of phenotypic diversification and adaptive evolution. One notable example is the identification of PKNOX2 as a key regulatory factor involved in mammalian cochlear development [24]. Additionally, convergent patterns of accelerated CNE evolution have been observed in hibernating lineages [25], underscoring the functional relevance of non-coding sequence divergence in specialized adaptations. To explore how shifts in evolutionary rates align with the emergence of specific phenotypic traits and deviations from molecular clock expectations, various computational methods have been developed to identify CNEs undergoing accelerated evolution, including PhyloP in PHAST [26], PhyloAcc [27], ForwardGenomics [28, 29], and ReRange [30, 31]. Despite these advancements, existing tools remain limited in scope. Most are restricted to detecting regions with general mutational enrichment, without the resolution to pinpoint specific, highly variable sites within CNEs. When evaluating accelerated CNEs, they only consider phylogenetic correlation factors, resulting in a high false negative rate. Moreover, they often require abundant dependency installation and multiple prerequisite steps, further complicating analysis and increasing computational burden. As a result, the discovery and functional implications of non-coding regulatory elements are considerably less advanced than our understanding of gene function [7, 8, 32]. Efficient and scalable identification of CNEs, along with robust assessment of their accelerated evolution, remains a major technical challenge.

A high-throughput and fully integrated bioinformatics toolkit, termed CNEwrap (Fig. 1B), was designed to enable the efficient identification of CNEs and prediction of their accelerated evolution across multiple genomes. Specifically, we developed a novel algorithm named “EvoAcc” to accurately detect accelerated evolving CNEs, which is implemented within the “evolve” subprogram of CNEwrap. This algorithm outperforms other existing tools in overall performance. Implemented primarily in Python, CNEwrap is freely available through both GitHub and Anaconda repositories. The CNEwrap toolkit and the EvoAcc algorithm are expected to promote future studies of CNEs in eukaryotic lineages.

## Materials and methods

### Input data preparation

Three types of input files are required for CNEwrap execution: (i) genome assemblies of the target species, together with a reference genome and its corresponding annotation file; (ii) a phylogenetic tree; and (iii) a configuration file. The configuration file specifies user-defined genetic distance parameters. To accommodate different levels of evolutionary divergence, CNEwrap provides three built-in divergence settings, designated as near, medium, and far, each corresponding to a distinct set of LASTZ parameters based on species divergence time and genetic distance. In the case studies presented here, the medium setting was used for all pairwise genome alignments.

### Whole-genome alignment

CNEwrap performs multi-genome alignment while minimizing information loss associated with reference genome bias. To improve alignment robustness and accuracy, the use of multiple reference genomes is recommended, and the union of aligned regions is retained to reduce the influence caused by genome assembly quality. The number of genomes included in each alignment block is dynamically adjusted based on the alignment results. Each reference genome is split into 20 segments (customizable) and aligned pairwise with all other genomes using LASTZ [19], with parallel computing enabling reduced alignment time. Detailed LASTZ usage has been described previously [33]. Each pairwise alignment is conducted using LASTZ under the phylogenetic distance setting (near, medium, or far) specified in the configuration file, with corresponding parameter details provided in Supplementary Table S1.

### Multiple alignment format manipulation

All pairwise alignments are converted into multiple alignment format (MAF) and merged using MULTIZ [21]. MAF blocks with alignment scores <20 000 are filtered using MafFilter [34]. The final MAF file is then split into single sequence alignments using the mafSplit function in UCSC-tools (http://hgdownload.cse.ucsc.edu/admin/exe/). When a phylogenetic tree is not provided, the integrated MAF alignment is further processed to generate full-sequence alignments or extract 4-fold degenerate (4D) sites, which are subsequently used for phylogenetic reconstruction with IQ-TREE [35]. Using 4D sites extracted from MAF alignment could produce a topological structure consistent with that of single-copy gene sets from OrthoFinder [36], along with very similar relative branch lengths (Supplementary Fig. S1).

### Conserved element scanning and identification

CNEwrap integrates GERP- and Phast-based methods for conserved element detection. GERP identifies regions exhibiting fewer substitutions than expected under neutrality and ranks constrained elements according to the inferred intensity of historical purifying selection [22]. The gerpelem module is executed with a depth threshold of 0.01. Phast predicts conserved elements using a maximum-likelihood phylogenetic hidden Markov model (phylo-HMM) [37]. Nucleotide substitution rates are modeled using REV [38], with parameters estimated by the expectation maximization (EM) algorithm and high-precision settings implemented in phyloFit. Conserved elements are then inferred with phastCons, using an expected conserved element length of 45 bp, a target coverage of 0.3 to fix transition rates, and a rho value of 0.3 to set the conserved-state evolutionary rate to 30% of the non–conserved state [23]. By combining the sensitivity of GERP to purifying selection intensity with the phylogenetic modeling capacity of Phast, including context-dependent substitution modeling and EM-based parameter estimation, this framework leverages their complementarity to achieve a more comprehensive detection of CNEs. Outputs from both methods are integrated using a custom script to take the union, and overlapping regions are reconciled to generate a unified set of high-confidence conserved elements. Merging is performed using BEDTools [39], permitting features located within 10 bp to be merged.

### CNE alignment construction and comparison

CNEwrap uses two Python-based modules, MergeBed and ExtractMAFbyBed, to assemble and compare CNE alignments. The MergeBed module consolidates GERP- and PhastCons-identified elements, excluding overlapping coding sequences (CDS) using the subtract function in BEDTools. The resulting non-coding elements are merged and sorted to generate a final non-redundant BED file of CNE coordinates. The ExtractMAFbyBed module extracts aligned sequences from genome-wide MAF files according to these coordinates. Contiguous alignment blocks are merged and annotated with CNE positional data to generate a concatenated MAF file. From this, target CNE sequences are exported in FASTA format, and associated GFF files containing coordinate information are generated for downstream analyses.

### Accelerated evolutionary estimation

Variations within conserved genomic elements are often associated with regulatory changes that drive functional divergence and phenotypic alterations [40, 41]. These variations can act as regulatory switches influencing the emergence of lineage-specific traits [42, 43].

To identify CNEs undergoing accelerated evolution, we developed a variation-scoring module in this study, termed EvoAcc. Accurate detection of species-specific accelerated variants from sequence alignments requires evaluating the conservation, positional specificity, and regulatory relevance of candidate loci and their flanking sequences. The EvoAcc algorithm quantifies these features to assess the conservation of these sites and their surrounding regions.

To account for phylogenetic structure and avoid confounding lineage effects, EvoAcc calculates the average evolutionary distance between designated foreground and background species, normalized by the maximum pairwise distance in the phylogeny. The average pairwise distance between foreground and background species is calculated as follows:


\begin{eqnarray*}
{{D}_{avg}} = \frac{1}{{| F| \cdot | B|}}\mathop \sum \limits_{f\in F} \mathop \sum \limits_{b\in B} d(f,b),
\end{eqnarray*}

where *F* and *B* represent the sets of foreground and background species, respectively, and *S* the set of all species in the tree.

Maximum distance between any two species is calculated using the following equation:


\begin{eqnarray*}
{{D}_{max}} = \mathop {max}\limits_{i,j\in S} \ d(i,j).
\end{eqnarray*}

The normalized group distance score, used to scale site-specific signal values, is determined using the following equation:


\begin{eqnarray*}
\textit{NormDist} = \frac{{{{D}_{avg}}}}{{{{D}_{max\ }}}}.
\end{eqnarray*}

To evaluate the conservation and fixation rate of variation sites, EvoAcc calculates the conservation of each alignment column using a Shannon entropy-based approach, as defined by Schneider and Stephens [44]. Information content (*IC*) is defined as:


\begin{eqnarray*}
IC = 2 + \sum \limits_{a\in A} p(a) \cdot {\log}_{2} (p(a)),
\end{eqnarray*}

where *A* represents a set of residues in the alignment column and *p(a)* represents the frequency of residue *a*. This metric quantifies sequence conservation, with higher values indicating greater constraint.

*IC_score* quantifies stable divergence between the foreground and background groups using Jaccard distance and weighted conservation scores:


\begin{eqnarray*}
J = 1 - \frac{{| {A \cap B}|}}{{| {A\cup B}|}},
\end{eqnarray*}

where *A* and *B* are the sets of unique residues in the foreground and background, respectively.


\begin{eqnarray*}
IC\_score = J \cdot \frac{{I{{C}_{fg}}}}{{I{{C}_{fg}} + I{{C}_{bg}}}},
\end{eqnarray*}

where ${\boldsymbol{I}}{{{\boldsymbol{C}}}_{{{\bf fg}}}}$, ${\boldsymbol{I}}{{{\boldsymbol{C}}}_{{{\bf bg}}}}$ are information content values for foreground and background, respectively.

To further assess the difficulty of base mutations between foreground and background residues, we calculate a weighted average nucleotide substitution distance using a log-odds matrix *M* calculated from the whole-genome alignments.

Let *B* be the set of nucleotides observed in the background alignment column, and let *p_b_* denote the relative frequency of base *b* within that background. For the foreground column, denote the nucleotides by *x_1_*, …, *x*_n_. Given the scoring matrix *M*, where *M(b,x)* represents the substitution score from base *b* to base *x*, we first compute the mean substitution score from each background base *b* to the foreground nucleotides:


\begin{eqnarray*}
{\overset{\leftharpoonup}{S}}_{b} = \frac{1}{n} \sum \limits_{i = 1}^n M( {b,{{x}_i}}).
\end{eqnarray*}

The background-weighted expected substitution score is then


\begin{eqnarray*}
{\overset{\leftharpoonup}{S}} = \sum \limits_{b\in B} {{p}_b}{{\overset{\leftharpoonup}{S}}}_{b}.
\end{eqnarray*}

The expected self-score of the background is


\begin{eqnarray*}
{{S}_{\textit{self}\ }} = \sum \limits_{b\in B}{{p}_b}M( {b,b}),
\end{eqnarray*}

and let *p_b_* denote the relative frequency of base. Finally, we define the difficulty index for substitution as


\begin{eqnarray*}
D = {{S}_{\textit{self}\ }} - \mathop S\limits^{\leftharpoonup} .
\end{eqnarray*}

If the foreground composition matches the background exactly, *D* = 0. Larger values of *D* indicate lower substitution from background to foreground.

Finally, the *acc_score* of sites is calculated as follows:


\begin{eqnarray*}
acc\_score = \frac{{D \cdot IC\_score}}{{\textit{NormDist}}}.
\end{eqnarray*}

The significant *acc_score* of variable sites is inferred by fitting a gamma distribution. A hypergeometric test is used to estimate the significance of enriched mutated sites in CNEs, flagged as putatively accelerated in the foreground species.

In parallel, two additional predictive tools can be optionally installed and incorporated into the pipeline. ForwardGenomics associates phenotypic variation with underlying genetic changes by identifying genomic elements that are consistently lost in lineages where specific traits have independently disappeared [28, 29]. PhyloP complements this by assigning statistical significance to conservation or acceleration at individual sites based on deviations from a neutral phylogenetic model [26]. The outputs from EvoAcc and ForwardGenomics or PhyloP are then combined to generate a unified, nonredundant set of CNEs exhibiting evidence of accelerated evolution.

### Comparison of four algorithms for accelerated evolution on simulation datasets

Simulation datasets developed for reptile species (Supplementary Fig. S2) were used to evaluate and compare the accuracy of different methods for detecting accelerated evolution in CNEs. The simulation approach followed previous research [27]. The parameters of the Gamma priors for substitution rates were set as follows: for positive classifications (non-conserved), the parameters were set to *nprior_a* = 15 and *nprior_b*= 0.1; for negative classifications (conserved), the parameters were set to *cprior_a* = 5 and *cprior_b* = 0.04. In both cases, the parameters *prior_a* and*prior_b*correspond to the alpha and beta values of the Gamma distribution, respectively. These datasets encompassed three scenarios involving substitution rate acceleration: a single acceleration, two independent accelerations, and three independent accelerations. The evaluation dataset comprised 500 simulated data entries, each 200 bp in length and extracted from reptile lineages forming a paraphyletic clade. PhyloAcc, PhyloP, and ForwardGenomics algorithms were selected as the benchmarks for comparison against our EvoAcc algorithm. Performance was assessed using the area under the precision-recall curve (AUPRC), calculated across varying ratios of conserved to accelerated loci, ranging from 1:1 to 100:1. For CNEwrap, classification accuracy was measured by the *acc_score*, which distinguishes between positive and negative predictions; for PhyloAcc, accuracy was estimated using Bayes Factor 1 (BF1); for PhyloP, accuracy was assessed using the *lnlratio*; and for ForwardGenomics, accuracy was evaluated using the *weightedPearsonCorrelationCoeff*. In addition, the computational resource consumption of EvoAcc and comparator algorithms was evaluated using progressively larger simulated datasets, including 500, 1000, 2000, 4000, 5000, and 8000 sequences, each 200 bp in length. All benchmarking analyses were performed on the same computer, with each test repeated at least three times and additional repetitions conducted when performance fluctuations were observed.

### Case studies of CNEwrap on real genomic datasets

Genome assemblies from three vertebrate lineages of fish, reptiles, and mammals (Supplementary Table S2) were used to show the overall performance of CNEwrap. Genome-wide CNE distributions were visualized in CNEwrap using a custom R script in the R package RIdeogram [45]. Subsequently, the following species were selected as the foreground branch to identify accelerated CNEs: for fish, *Anabas testudineus* (Ates) and *Boleophthalmus pectinirostris* (Bpec); for reptiles, *Thermophis baileyi* (Tbai) or *Thamnophis elegans* (Tele); and for mammals, *Homo sapiens* (Hsap). The phylogenetic tree of reptile species was reconstructed based on prior comparative genomics analysis [46]. Method-specific significance thresholds were set as follows: *P*-value < .01 for EvoAcc; *P*-value from −.01 to .01 for PhyloP; *P*-value < .05 for ForwardGenomics (due to its more stringent calculation); and logBF1 ≥ 1 and logBF2 ≥ 1 for PhyloAcc. To further assess robustness of EvoAcc under phylogenetic uncertainty, additional trees were generated with randomly varying branch lengths and topologies, together with bootstrap trees generated with RAxML [47], using the reptilian phylogeny as the template. Relative variability of *acc_score* across trees was quantified using the coefficient of variation (CV), a standard statistic for measuring relative dispersion [48]. Directional consistency across trees was further incorporated to define a composite robustness score ranging from 0 to 1 by integrating variability and sign agreement. Genes located within 200 kb of each CNE were designated as putative regulatory targets. CNEwrap provides R scripts to visualize the specific chromosomal locations and sequence mutation or InDel of CNEs of interest in the tested species by querying their unique IDs. Moreover, to evaluate information loss associated with reliance on a single reference genome, CNEs were identified separately using Tbai and Tele as reference genomes, and the union of both sets was used as the final integrated result.

### Validation of the EvoAcc algorithm

To validate performance of EvoAcc, we downloaded the location information of 10 032 hCONDELs in humans from a previous study [49]. Following their original method (the species-specific activity BH-adjusted *P*-value <.05 and the activity BH-adjusted *P*-value in the human or chimpanzee sequence <.1, after excluding hCONDELs with low representation), we collected 803 hCONDELs with species-specific regulatory effects. The accelerated evolution status of each hCONDEL was predicted based on our mammalian dataset using the four algorithms to determine whether the predictions aligned with the experimentally validated hCONDELs. Additionally, we collected a dataset of 1363 human accelerated regions (HARs) and 3027 human-gained enhancers [50], of which 532 exhibited human-specific regulatory activity. This dataset was subjected to the same validation analysis described earlier.

## Results

### CNEwrap and the EvoAcc algorithm involved in “evolve” subprogram

To efficiently identify CNEs from large-scale multi-species genomic datasets spanning broad evolutionary distances, together with systematic detection of accelerated evolution, we developed an integrated pipeline named CNEwrap. The CNEwrap pipeline comprises five modular subprograms, including align, merge, scne, evolve, and trace (Fig. 1B), which collectively streamline the workflow from genome alignment to evolutionary analysis. The five core modules perform the following tasks: whole-genome alignments (capable of parallel execution); consolidation of multiple alignment format (MAF) files; scanning for CNEs; deduplication of CNEs, followed by filtering out CDS overlaps and performing trace alignment; and finally, detecting evolutionary selection patterns using a newly developed EvoAcc algorithm introduced in this study. A detailed description of the CNEwrap workflow is provided in Supplementary Text 1.

**Figure 2.**
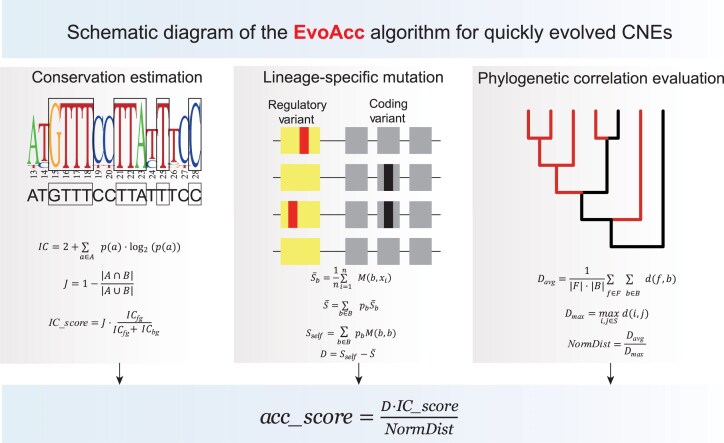
The three main components of the EvoAcc algorithm for evaluating accelerated evolving CNEs: conservation evaluation, mutation-specific evaluation, and phylogenetic correlation evaluation.

EvoAcc is implemented within the “evolve” subprogram of this framework, and is designed to quantify mutational dynamics based on integrating sequence conservation and information content at specific genetic loci (Fig. 2). First, EvoAcc calculates the conservation of each alignment column using a Shannon entropy-based approach to derive the *IC_score*. Second, it quantifies a weighted average nucleotide substitution distance between the foreground and background groups based on a log-odds matrix *M* to get the *D* values. Third, it computes the average pairwise distance and the normalized group distance to assess the phylogenetic correlation among species, yielding the *NormDist*. By considering these three aspects, EvoAcc develops an *acc_score* to assess the mutational dynamics for each CNE. In contrast to existing methods that primarily emphasize phylogenetic correlation, EvoAcc incorporates multiple dimensions of sequence variation and therefore improves detection of sites with potential lineage-specific acceleration. A detailed description of the EvoAcc workflow and operational logic is provided in Supplementary Text 2.

### Accuracy assessment of accelerated evolution detection with simulation datasets

**Figure 3.**
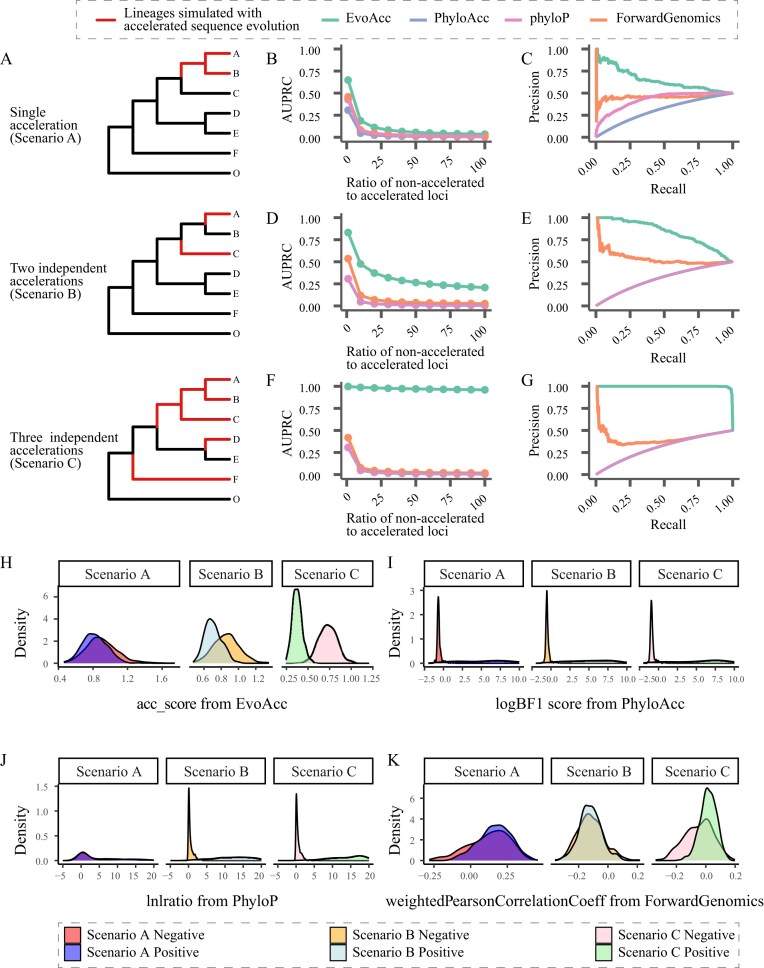
Accuracy comparison between the “evolve” subprogram in EvoAcc and three well-known algorithms using simulated datasets based on reptile phylogeny. (**A**) Three evolutionary scenarios were tested: a single monophyletic acceleration, two independent accelerations, and three independent accelerations. (**B**), (**D**), and (**F**) show AUPRC values across varying ratios of conserved to accelerated loci. (**C**), (**E**), and (**G**) show precision-recall curves under a fixed ratio of 50 conserved loci for each accelerated locus. (**H**), (**I**), (**J**), and (**K**) show distributions of *acc_score* from EvoAcc, *logBF1* from PhyloAcc,*lnlratio* from PhyloP, and *weightedPearsonCorrelationCoeff* from ForwardGenomics for positive and negative classifications across scenarios A, B, and C.

To evaluate CNEwrap performance on accelerated evolution detection, the EvoAcc algorithm within CNEwrap was compared with PhyloAcc, PhyloP, and ForwardGenomics using three simulation datasets representing distinct substitution rate acceleration scenarios (Fig. 3A): single acceleration (scenario A), two independent accelerations (scenario B), and three independent accelerations (scenario C). Computational benchmarks (Table 1) indicated that EvoAcc and PhyloP required substantially less CPU time and lower CPU usage than ForwardGenomics and PhyloAcc. Among all evaluated methods, EvoAcc exhibited the slowest increase in memory consumption as the number of simulated sequences increased. Similar benchmarking trends were observed across all three evolutionary scenarios and both classification types (Supplementary Tables S3 and S4). Across all scenarios, EvoAcc maintained higher predictive accuracy, with AUPRC values for EvoAcc exceeded those of other algorithms ranging from 15% to 95% (Fig. 3B, D, and F). At comparable recall levels, EvoAcc also achieved superior precision (Fig. 3C, E, and G). In scenarios B and C, EvoAcc showed more significant advantages compared with other algorithms. These findings indicate that EvoAcc offers the best overall performance in accuracy assessment, particularly in detecting lineage-specific accelerated substitutions when more lineages are involved.

**Table 1.** Comparison of CPU time, CPU usage, and memory usage between EvoAcc and other algorithms under positive classifications across scenario A

		Simulated sequences number					
Algorithm	Resources occupancy	500	1000	2000	4000	5000	8000
EvoAcc	CPU Time	0 m 45 s	1 m 22 s	2 m 39 s	5 m 11 s	6 m 28 s	9 m 25 s
	CPU Usage (%)	99.9	100.9	100.9	100.9	100.9	100.9
	Memory Usage (Gb)	12.2	13.8	17	23.5	26.4	35.9
PhyloAcc	CPU Time	24 m 34 s	33 m 23 s	73 m 20 s	124 m 55 s	156 m 3 s	254 m 39 s
	CPU Usage (%)	1004.1	2006.1	2563.7	2833	2831	2813
	Memory Usage (Gb)	14.3	24.2	44.1	84.1	103.6	163.3
PhyloP	CPU Time	0 m 35 s	1 m 3 s	2 m 6 s	4 m 5 s	5 m 5 s	7 m 29 s
	CPU Usage (%)	100.9	101.9	100.9	100.9	100.9	100.9
	Memory Usage (Gb)	3.7	8.3	10.5	25.5	40.3	70.5
ForwardGenomics	CPU Time	1 m 34 s	2 m 26 s	4 m 41 s	10 m 26 s	12 m 25 s	20 m 54 s
	CPU Usage (%)	1600.4	1600.2	1600.9	1600.9	1601.2	1601.2
	Memory Usage (Gb)	42.3	39.4	47.5	51.4	53.7	53.2

*Verified by multiple replicates, the non-monotonic trend for ForwardGenomics stems from the underlying R environment, where memory fragmentation patterns fluctuate depending on unique sequence characteristics.

CPU usage is reported as a percentage relative to a single core. Values of CPU usage exceeding 100% indicate parallel multi-core utilization.

Discriminatory power was further evaluated by comparing the Gamma priors of negative and positive classifications. EvoAcc showed clearly separated distributions for the two classifications in scenarios B and C, and although overlap was observed in scenario A, the peaks corresponding to the *acc_score* remained distinct and separable (Fig. 3H). In contrast, ForwardGenomics displayed considerable overlap in both the data distributions and the peaks corresponding to the weightedPearsonCorrelationCoeff, reducing classification clarity (Fig. 3K). Furthermore, the distributions of PhyloAcc and PhyloP show significant deviations from the simulated data (Supplementary Fig. S3), resulting in less reliability of their results (Fig. 3I and J). Overall, EvoAcc achieved more accurate and computationally efficient detection of accelerated CNEs, particularly under conditions involving two or three accelerated lineages.

### Case studies of CNEwrap performance in cross-species and accelerated CNE identification

**Figure 4.**
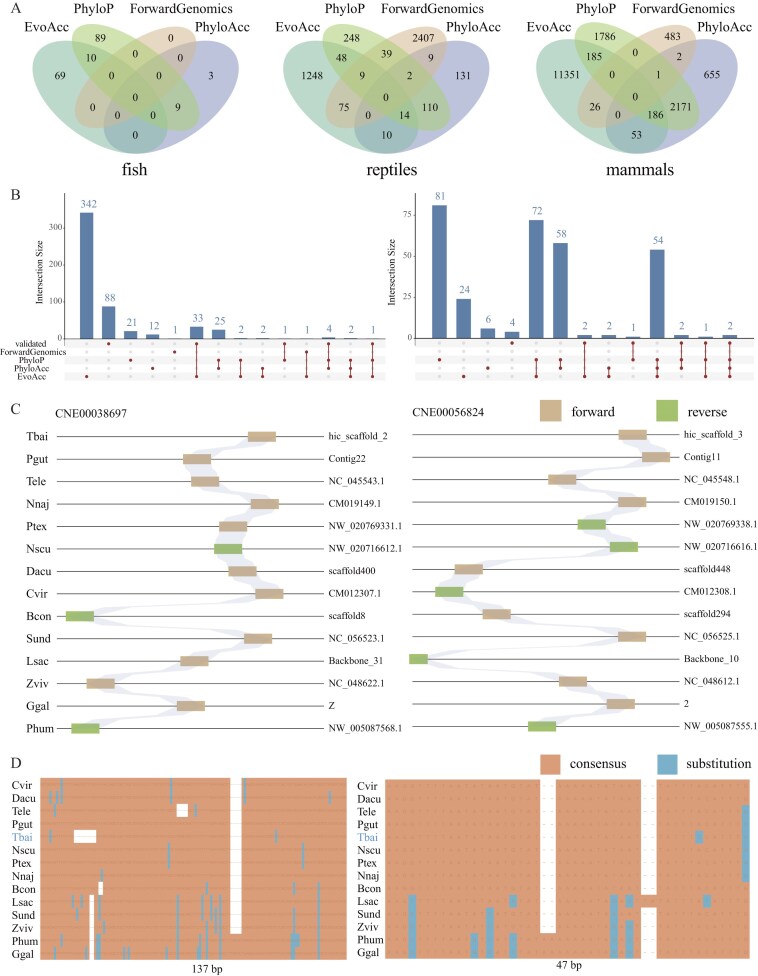
The case studies of accelerated CNEs detected by EvoAcc. (**A**) The comparison of four algorithms applied to real genomic data of fish, reptiles, and mammals. (**B**) Performance assessment on the validated human hCONDELs dataset. The red dots represent the counts for each method category. (**C**) Chromosomal synteny of the accelerated CNEs identified by EvoAcc across tested reptile species. (**D**) Multiple sequence alignments of CNE00038697 and CNE00056824 in tested reptile species, with *Thermophis baileyi* (Tbai) as the foreground branch.

CNEwrap performance was evaluated using real genomic data from three vertebrate lineages of fish, reptiles, and mammals (Supplementary Fig. S4 and Supplementary Table S2). Across reptilian species, 30 757 CNEs were identified after removal of 15 882 CDS–overlapping elements. In fish, 1934 CNEs were retained following exclusion of 5135 CDS–overlapping elements. In mammals, 251 221 CNEs were identified after removal of 21 503 CDS–overlapping elements. To evaluate the performance of the EvoAcc algorithm in detecting accelerated CNEs from real genomic dataset, we identified accelerated CNEs across all three lineages (Fig. 4A). In the three lineages, EvoAcc identified a relatively large number of accelerated CNEs in each lineage, with the highest number observed in mammals. Manual inspection of mammalian accelerated CNEs further showed that EvoAcc detected identifiable lineage-specific InDel events and nucleotide substitutions in the foreground branch, which were not recovered by the other algorithms (Supplementary Fig. S5). For further validation, we overlapped the collected hCONDELs with our mammalian dataset. 1701 CNEs were mapped on hCONDELs, and 127 were mapped on the validated hCONDELs with species-specific regulatory effects. Among these validated hCONDELs, EvoAcc, PhyloAcc, PhyloP, and ForwardGenomics identified 34, 4, 6, and 0 accelerated CNEs, respectively (Fig. 4B left). As most hCONDELs are human-specific deletions, EvoAcc’s specific optimization for InDel scoring likely contributes to its competitive advantage. The second similar validation was performed using a dataset of HARs and human-gained enhancers, from which 352 elements were mapped to our mammalian dataset and 12 exhibited human-specific regulatory activity. In this set, EvoAcc, PhyloAcc, PhyloP, and ForwardGenomics detected 5, 4, 6, and 0 validated accelerated CNEs, respectively (Fig. 4B right). Collectively, these results indicate that EvoAcc performs at least comparably to existing methods, while also providing complementary detection capacity for accelerated CNEs.

Robustness of EvoAcc was further evaluated under variable branch lengths and topological configurations using randomly generated alternative trees and bootstrap trees derived from RAxML, all based on the reptilian phylogeny and dataset. Robustness exceeded 80% under random perturbation scenarios and reached 90% in bootstrap analyses. More specifically, when only branch lengths were randomly varied relative to the input tree, the robustness score was 0.8726 [95% confidence interval (CI): 0.8725919–0.8725921]. When topology alone was randomly varied, the robustness score was 0.8284 (95% CI: 0.8283886–0.8283890). Across all randomly generated alternative trees, the robustness score was 0.8753 (95% CI: 0.8753067–0.8753069). For bootstrap trees, the robustness score reached 0.9063 (95% CI: 0.9062940–0.9062943).

Accelerated CNEs uniquely identified by EvoAcc in reptile species were further used as representative examples for visualization. The generated outputs included precise genomic positions for each CNE across the analyzed species (Fig. 4C), enabling direct sequence extraction and downstream investigation of potential interspecies variation (Fig. 4D). Accurate genomic positioning also facilitated identification of putative target genes in flanking regions. For example, two lineage-specific accelerated CNEs (*acc_score P*-value <.01), CNE00038697 and CNE00056824, were identified near the excision repair cross-complementation group 6-like 2 (*ERCC6L2*) gene and the transient receptor potential ankyrin subtype 1 (*TRPA1*) gene, respectively. These accelerated CNEs may influence gene regulation in Tbai, which contributes to its DNA damage repair [51] and cold adaptation [46]. Furthermore, transcription factor binding sites (TFBSs) annotation process can be performed on the final accelerated CNEs dataset to understand their potential regulatory functions and evolutionary preferences. This framework connects the regulatory cascade from CNE variation to gene regulation and downstream biological processes, benefiting further mechanistic studies.

To assess information loss associated with the use of a single reference genome, genome-wide comparisons were performed using both the Tbai and Tele reference genomes (Supplementary Fig. S6). In the same set of tested species, 46 639 CNEs were identified using Tbai, and 44 537 CNEs were identified using Tele. The number of shared CNEs accounted for 83.12% of the total CNEs identified using Tbai and 85.06% of those identified using Tele. The combined use of both reference genomes recovered an additional 15%–17% of CNEs, highlighting the value of multi-reference strategies for maximizing CNE detection across species.

## Discussion

CNEs constitute a widely distributed class of genomic sequences detectable across diverse evolutionary lineages. Accumulating evidence has revealed that CNEs are functionally engaged in a broad variety of regulatory processes (Fig. 1A), positioning them as central components in the study of genome evolution and gene regulation. The functional roles of CNEs subject them to natural selection pressure, leading to a tendency toward evolutionary conservation. The EvoAcc algorithm, which takes into account nucleotide variation frequencies and phylogenetic relationships among species while reconciling global conservation and clade-specific divergence, offers insights of greater evolutionary relevance. In contrast, PhyloP and PhyloAcc primarily emphasize the accumulation and intensity of sequence variation, whereas ForwardGenomics focuses on the relationship between genetic variation and phenotypic traits, potentially providing a more comprehensive evaluation framework.

CNEwrap toolkit and EvoAcc algorithm address long-standing limitations in the field by providing a robust, high-throughput pipeline for the systematic identification of CNEs and detection of sites undergoing accelerated evolution based on multiple genome alignments. By consolidating previously labor-intensive and fragmented analytical steps into a unified command-line framework, the CNEwrap toolkit and the EvoAcc algorithm significantly advance the scalability and accessibility of CNE-focused genomic research.

CNEwrap enhances CNE identification across four principal domains. First, the “align” module incorporates an optimized genome alignment strategy guided by phylogenetic distance matrices to maximize alignment depth across taxonomic groups. This approach improves both sensitivity and coverage in cross-species CNE detection. Given the substantial influence of reference genome quality on alignment outcomes, the use of two reference genomes is recommended, with the union of both results retained as the final integrated set. This dual-reference strategy effectively mitigates information loss caused by large-scale deletions and improves detection accuracy. Nonetheless, large-scale insertions or structural variants present in query genomes may still be missed or poorly aligned. Future improvements may therefore benefit from pangenome- or graph-based frameworks, as well as assembly-based comparative approaches, which may enhance alignment quality and improve recovery of lineage-specific CNEs. Furthermore, for high-performance computing environments, the pipeline supports Sun Grid Engine (SGE)-based job scheduling to accelerate large-scale alignments.

Second, CNEwrap achieves superior performance in identifying accelerated evolutionary signals within CNEs. We developed EvoAcc, a custom algorithm designed to detect base-level divergence using entropy-based conservation metrics and mutation distance matrices. Compared with other algorithms on simulation datasets, EvoAcc delivers the highest precision and sensitivity, particularly in scenarios involving two or three accelerated lineages. This suggests that EvoAcc is especially effective in detecting evolutionary rate shifts in phylogenetically broader clades. When acceleration events are only distributed across a single lineage, EvoAcc still outperforms the other three algorithms, but the accuracy of all methods is decreased because it becomes harder to detect them owing to the smaller number of accelerated clades. The second-best performer is the ForwardGenomics algorithm; however, its high computational resource requirements and complex dependency installation make it relatively difficult to employ in practical applications. On real genomic data, EvoAcc shows a comparable hCONDELs positive rate to other methods, yet yields a larger number of predictions. The underlying design of EvoAcc also contributes to its enhanced sensitivity for acceleration driven by InDel events. Notably, EvoAcc captures identifiable InDels and nucleotide substitutions between foreground and background branches that are frequently missed by other algorithms. This sensitivity arises from the combined contributions of *IC*, which imposes stringent alignment consistency, and *D*, which weights base-level substitutions and InDels according to a base substitution matrix inferred from whole-genome alignments. Because InDels occur less frequently than single nucleotide variants yet often exert larger functional effects, EvoAcc improves sensitivity for detecting this class of variation. This pattern is biologically plausible, as InDels often produce larger effects on gene expression than nucleotide substitutions [52–54]. In contrast, other algorithms primarily emphasize overall evolutionary rates and are less sensitive to the contributions of individual InDel events. Collectively, these findings indicate that EvoAcc serves as a valuable complementary tool for obtaining a more comprehensive set of accelerated CNEs. When computational resources allow, combined application of EvoAcc with other methods is recommended for accelerated evolution analysis.

Furthermore, the modular design of EvoAcc also allows for future extension beyond its current application. Among its three core parameters, *IC_score* and *D* can be adapted for different data types and taxonomic groups. *IC_score* is designed for groups with close relationships, such as pangenomes, which are characterized by very small genetic distances and thus naturally compatible with the current scoring scheme. Meanwhile, the *acc_score* module could be extended to coding sequences by scanning these regions with a suitable DNA substitution matrix, offering a powerful complement to existing codon-based selection detection methods.

Third, CNEwrap offers a fully automated, end-to-end workflow that integrates all core steps of CNE identification. Written in Python and designed with modular integration, the pipeline is easily installed and maintained across diverse computing environments. All stages—including whole-genome alignment, data merging, CNE identification, and evolutionary rate analysis—can be executed through a single command, representing a substantial advancement in user efficiency and reproducibility. Moreover, the output is extensive and highly informative, providing precise genomic coordinates for each identified CNE, corresponding multiple sequence alignments, and visualization scripts for clear presentation of the results. Accurate genomic coordinates allow comparison of putative target genes associated with the same CNE across species. Because these target genes may differ among lineages, identifying variations from sequence alignments further supports investigation of potential functional divergence in the corresponding CNEs.

Finally, EvoAcc retains strong robustness under phylogenetic uncertainty. In phylogenomic analysis, systematic errors in inferred relationships remain a persistent challenge. Missing data and poor taxon sampling can produce long-branch attraction artifacts, while incomplete lineage sorting, hybridization, and horizontal gene transfer can further distort underlying phylogenetic structure [55]. Methods that infer evolutionary rate shifts from phylogenetic relationships, including PhyloAcc, are likewise vulnerable to these sources of error [56]. In contrast to approaches that rely primarily on phylogenetic correlation, EvoAcc integrates two additional dimensions for detecting evolutionary rate shifts, namely conservation evaluation and mutation-specific evaluation. This multi-component framework increases resilience to phylogenetic errors. EvoAcc maintained robustness scores above 80% across analyses with randomly varying branch lengths and topologies, and robustness approached 90% when bootstrap trees were used.

Despite these advantages, certain limitations exist. Notably, the identification of accelerated evolution may be confounded by local genomic architectures and heterogeneous evolutionary rates. When integrating results from multiple methods for comparative evolutionary analysis, the impact of phylogenetic tree structure and branch length heterogeneity on detection bias should be carefully considered. We recommend selecting species sets with relatively balanced branch lengths to reduce systematic bias across methods caused by tree imbalance. If an unbalanced tree is unavoidable, the direction breakdown (acceleration/conservation ratio) of each method’s results should be reported alongside. Another limitation is that in the case of deeply divergent lineages, such as teleosts, homologous non-coding elements often exhibit extensive sequence divergence [57], limiting the effectiveness of alignment-based detection methods. As a result, the number of detectable CNEs declines substantially in such comparisons. Although CNEwrap facilitates cross-lineage detection sensitivity, it remains constrained by this fundamental limitation. Emerging alignment-free approaches, particularly those based on deep learning, offer an alternative framework for CNE prediction [58, 59]. However, these methods currently suffer from high false-positive rates and limited interpretability, underscoring the need for further refinement. Continued technological advancements are expected to address current limitations in identifying CNEs across highly diverged evolutionary clades.

## Supplementary Material

gkag709_Supplemental_Files

## Data Availability

CNEwrap, along with all case study code used in this research, is available via the GitHub repository (https://github.com/YanCCscu/CNEwrap) and the FigShare dataset (DOI: 10.6084/m9.figshare.29625779).
