# Supplementary material for: CNEwrap: a scalable toolkit with a novel algorithm for large-scale genome-wide accelerated conserved non-coding elements detection
Source: Nucleic Acids Res. 2026 Jul 20;54(14):gkag709. doi: 10.1093/nar/gkag709 (PMC13384253; doi:10.1093/nar/gkag709)
Supplement: gkag709_Supplemental_Files [file gkag709_supplemental_files.zip › Supplementary_File26.4.21.docx]

**Supplementary Files**

**Supplementary Text 1** The workflow of CNEwrap pipeline.

**Input files**

1. Genome sequences of selected species in **FASTA** format (including target and reference genome files; file name suggested as species name or abbreviation), and the reference genome requires the corresponding annotation file in **GFF** format.

2. A species tree file in **Newick** format containing all the selected species, with node names matching genome file names.

3. Phylogenetic relationship table **lastz.dist** (optional, default: medium). Three columns: reference species, target species, relationship keyword (near/medium/far). Can be generated by TreeManipulation.py and manually edited if needed.

**Module1: align**

Preprocesses genomes and performs whole-genome alignment. **Maps each target genome to the reference**. For large references, run RepeatMasker and split reference into parts (-n) for parallel processing. The **tree file** specifies the genomes for alignment to the reference, no manual specification is required. **lastz.dist** provides Lastz parameters. Output: **MAF alignment files** from the Lastz pipeline (axt → axt2chain → sortchain → chainnet → netfilter → net2axt → axt2maf).

**Module2: merge**

Integrates pairwise MAFs and performs quality control. The **MAF alignment files** produced from align module are merged to a merged MAF file using multiz, guided by phylogenetic distance from the reference (closest to farthest). The merged MAF file is quality controlled with parameters of -minRow={species_number} -maxRow=500 -minCol=30 -minScore=20000. Finally, the merged and filtered MAF file is further split into separate MAF files by block. Output: **Block-split MAF files**.

**Module3: scne**

Scans CNEs from the **Block-split MAF files** obtained from the merge module. CNEs are identified using GERP and Phast tools. Output: **BED files** obtained from GERP and Phast, containing genomic positions information and conservation scores of conserved regions.

**Module4: trace**

Extracts the corresponding sequence alignments from CNEs. The **BED files** obtained from GERP and Phast are integrated to merge overlapping regions, excluded coding sequences based on **GFF** file of reference genome, and filtered the CNEs don’t meet length requirements, resulting in **a final non-redundant BED file**. Based on the non-redundant BED file and the **Block-split MAF files** obtained from the merge module, the sequences and genomic positions of target CNEs are extracted. Outputs: **Multiple sequence alignments (FASTA)** and **Annotation file (GFF)**.

**Module5: evolve**

Analyzes evolutionary rates of CNEs based on **Multiple sequence alignments (FASTA)** that obtained from trace module. The accelerated CNEs are identified using EvoAcc, ForwardGenomics (FG) and PhyloP (PP).

**Supplementary Text 2** The pseudocode of EvoAcc algorithm.

EvoAcc code mainly: 1) detects accelerated evolution in DNA sequences for specific foreground species compared to background species; 2) evaluates the significance of acceleration using statistical methods; and 3) outputs site-level and gene-level acceleration results.

**# EvoAcc Algorithm Pseudocode**

**Input**：

alidir: directory containing alignment files

species: foreground species (comma-separated)

treefile: phylogenetic tree file

distfile: distance matrix file

bgfile: background species file (optional)

**Output**：

Site-level detailed results

Gene-level summary results

**Steps**：

1. Initialization

- Set significance threshold SIG_THRESHOLD = 0.05

- Set distance constant K_dist = 1

2. Compute species group distances

- Read phylogenetic tree

- Separate foreground/background species

- Calculate average distance between groups

- Calculate maximum possible distance

- Compute normalized distance (***NormDist***) score

3. Parse distance matrix

- Read nucleotide distance matrix

- Convert to dictionary format for convenient query

4. Batch process alignment files

For each alignment file:

a. Read multiple sequence alignment

b. Build species index mapping

c. extract foreground/background species sequences

For each site:

1. Extract foreground/background sequence characters

2. Filter sites with excessive gaps

3. Compute information content:

- Foreground sequence IC (***IC_fg_***)

- Background sequence IC (***IC_bg_***)

- Overall sequence IC

4. Calculate Jaccard distance (***J***):

- Compare foreground/background nucleotide set differences

5. Calculate weighted distance (***D***):

- Based on distance matrix

- Account for special characters

6. Compute final acceleration score:

$\text{IC\_score}=J\cdot\frac{IC_{\text{fg}}}{IC_{\text{fg}}+IC_{\text{bg}}}$

$\text{acc\_score}=\frac{D\cdot IC\text{\_score}}{\text{NormDist}}$

5. Statistical testing

- Collect all site scores

- Fit data with gamma distribution

- Calculate *p-value* for each site

- Identify significant sites (*p-value* < 0.01)

6. Output results

a. Site-level output:

- Gene ID, site position, Jaccard score

- Information content values, weighted distance

- Acceleration score, *p-value*, signal type

b. Gene-level output:

- Sequence length, number of variable sites

- Number of significant sites, total score, mean score, peak score

- Gene-level *p-value* (hypergeometric test)

- Gene acceleration status

**Supplementary Figure 1** The construction of the reptile species trees using 4D sites extracted from MAF alignment (A) and single-copy gene sets from OrthoFinder (B).


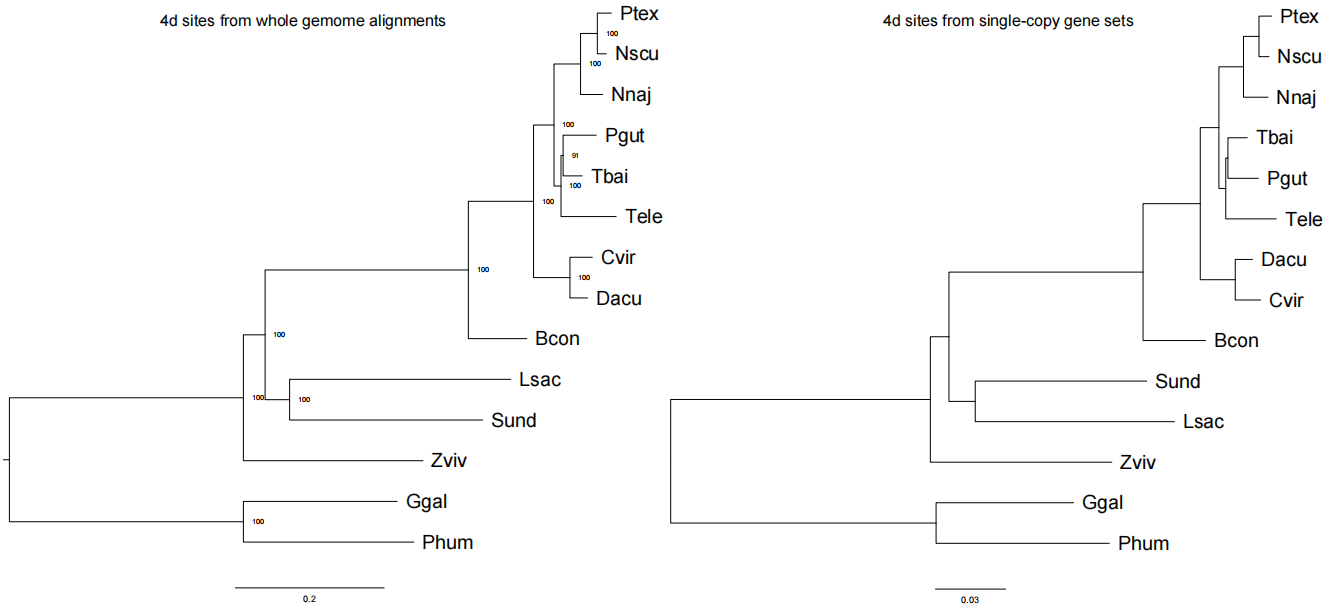


A

B

**Supplementary Figure 2** The phylogeny of a simulated reptile dataset with internal nodes (red) and tips (blue).


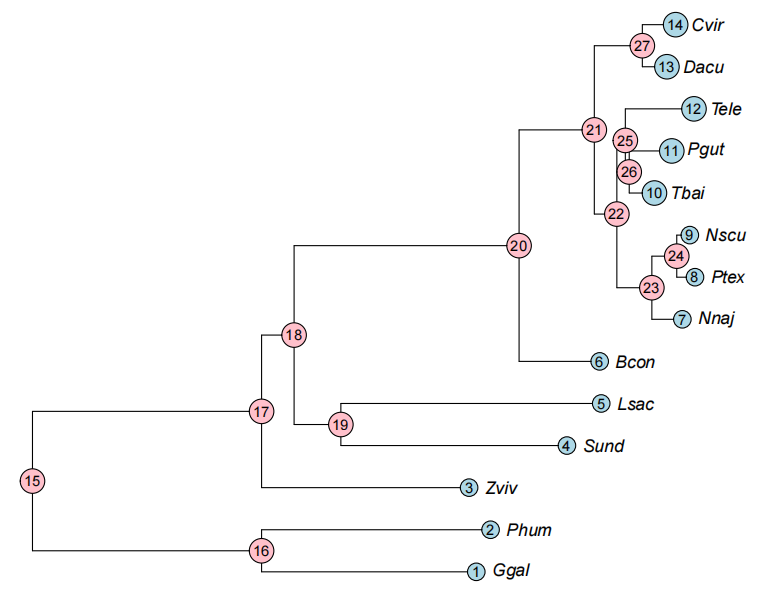


**Supplementary Figure 3** Gamma distributions for substitution rates using simulated data. Positive (non-conserved) and negative (conserved) classifications are indicated by the blue and red curves, respectively.

**
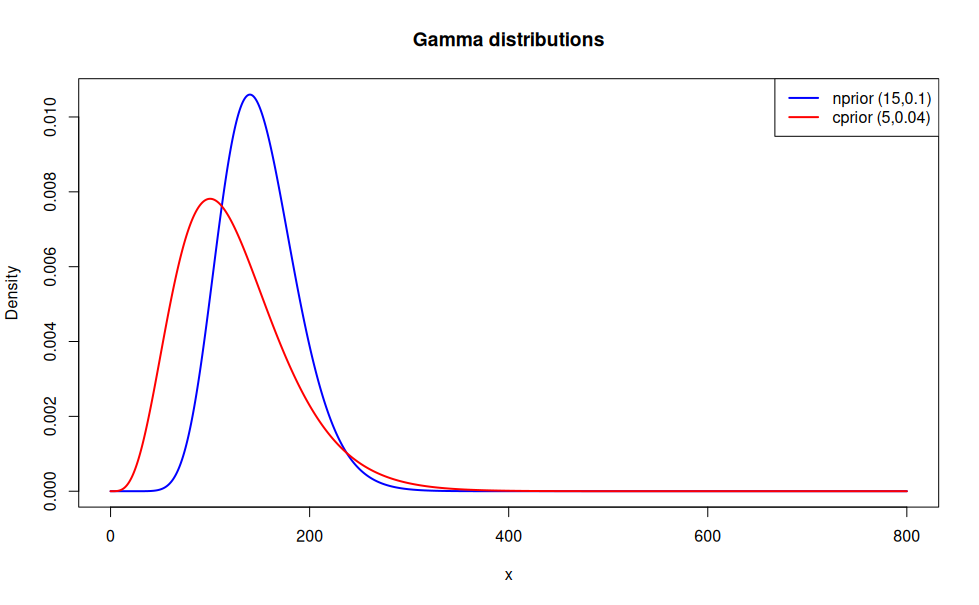
**

**Supplementary Figure 4** Genomic distribution of CNEs in vertebrate species: (A) *Thamnophis elegans* (Tele), (B) *Zootoca vivipara* (Zviv), (C) *Danio rerio* (Drer), (D) *Oryzias latipes* (Olat), (E) *Homo sapiens* (Hsap) and (F) *Mus musculus* (Mmus), identified using CNEwrap.


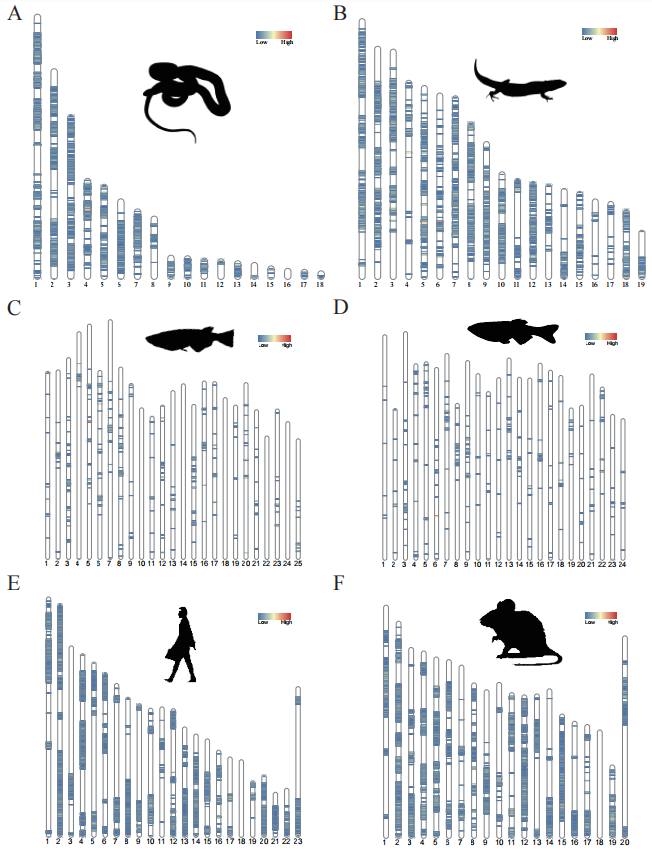


**Supplementary Figure 5** Multiple sequence alignments of the candidate unique accelerated CNEs detected by EvoAcc in the mammalian dataset, with Hsap as the foreground branch.


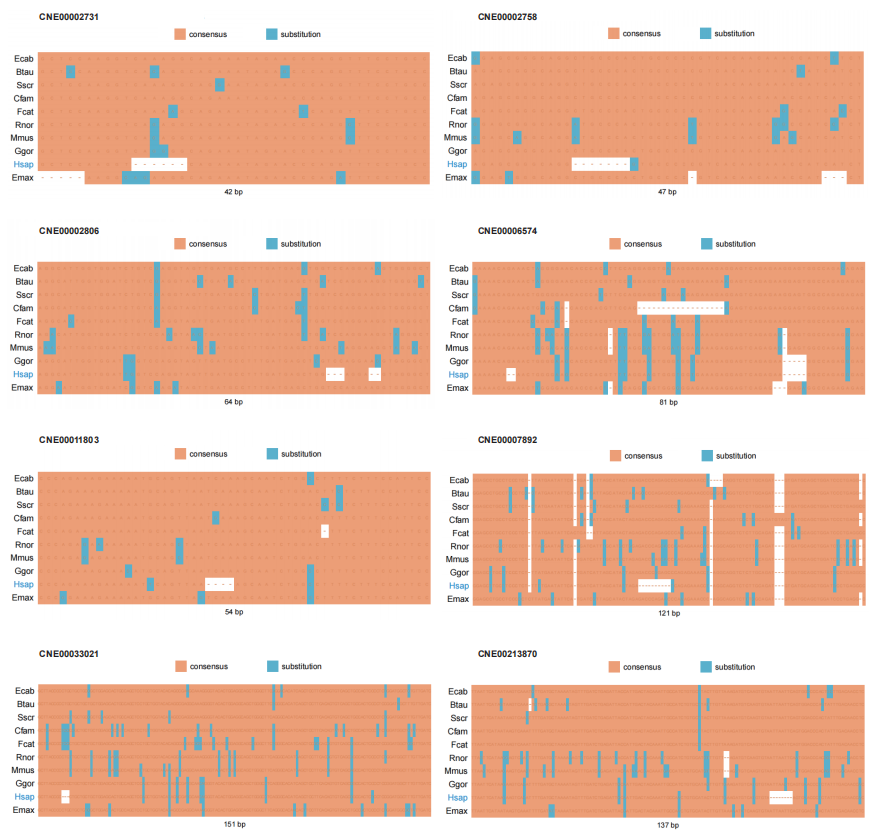


**Supplementary Figure 6** An example for comparing CNEs count and distribution between the two reference genomes.

**
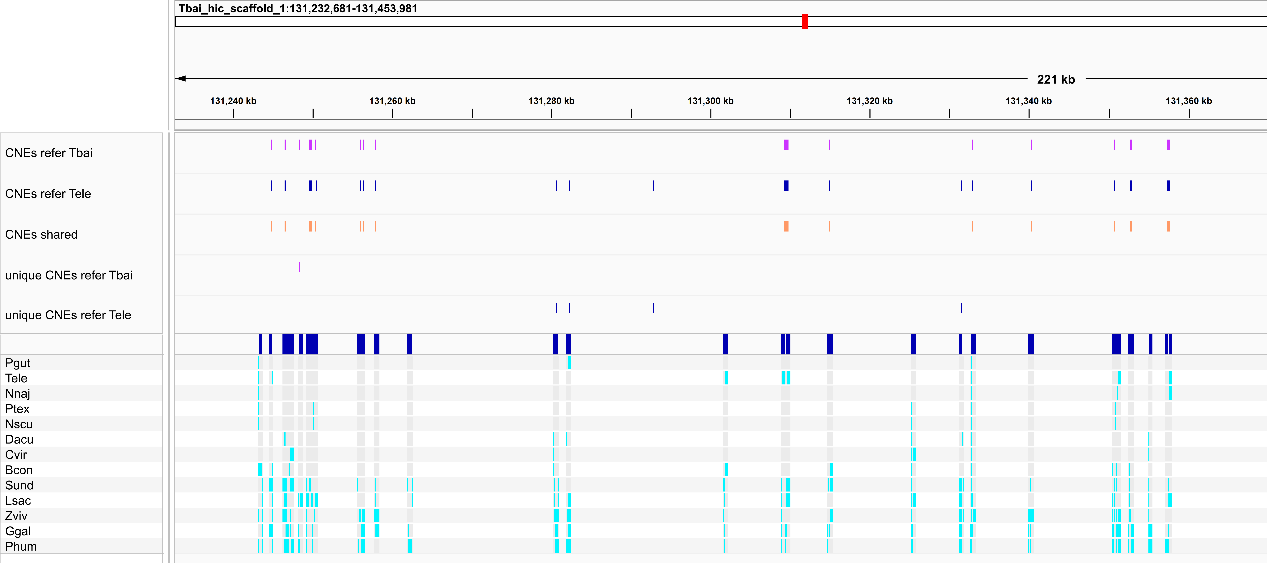
**

**Supplementary Table 1** Parameters of three built-in divergence settings (near, medium, and far) used in LASTZ for whole-genome alignment.

| LASTZ Parameter | Near | Medium | Far |
| --- | --- | --- | --- |
| Gap open penalty (O) | 600 | 400 | 400 |
| Gap extend penalty (E) | 150 | 30 | 30 |
| high-scoring segment pairs threshold (K) | 4500 | 3000 | 2200 |
| Threshold for gapped extension (L) | 2200 | 2200 | 6000 |
| Threshold for alignments between gapped alignment blocks (H) | 2000 | 2000 | 2000 |
| Masking count (M) | 254 | 50 | 50 |
| Seed and Transition value (T) | 2 | 1 | 2 |
| Threshold for terminating gapped extension (Y) | 15000 | 9400 | 3400 |
| Scoring matrix (Q) | near.q | medium.q | far.q |

Score matrix files “near.q”:

|  | A | C | G | T |
| --- | --- | --- | --- | --- |
| A | 90 | -330 | -236 | -356 |
| C | -330 | 100 | -318 | -236 |
| G | -236 | -318 | 100 | -330 |
| T | -356 | -236 | -330 | 90 |

Score matrix files “medium.q”:

|  | A | C | G | T |
| --- | --- | --- | --- | --- |
| A | 91 | -114 | -31 | -123 |
| C | -114 | 100 | -125 | -31 |
| G | -31 | -125 | 100 | -114 |
| T | -123 | -31 | -114 | 91 |

Score matrix files “far.q”:

|  | A | C | G | T |
| --- | --- | --- | --- | --- |
| A | 91 | -90 | -25 | -100 |
| C | -90 | 100 | -100 | -25 |
| G | -25 | -100 | 100 | -90 |
| T | -100 | -25 | -90 | 91 |

**Supplementary Table 2** Genome data used for performance assessment and CNEs database construction.

| Lineages | Deposited data | Abbreviation | Source | Accession NO./website |
| --- | --- | --- | --- | --- |
| Reptiles | *Thermophis baileyi* | Tbai | NGDC | GWHBJWY00000000 |
| Reptiles | *Pseudonaja textilis* | Ptex | NCBI | GCA_900608585.1 |
| Reptiles | *Pantherophis guttatus* | Pgut | NCBI | GCF_001185365.1 |
| Reptiles | *Crotalus viridis viridis* | Cvir | NCBI | GCA_003400415.2 |
| Reptiles | *Notechis scutatus* | Nscu | NCBI | GCF_900518725.1 |
| Reptiles | *Deinagkistrodon acutus* | Dacu | GigaBase | ftp.cngb.org/pub/gigadb/pub/10.5524/100001_101000/100196/ |
| Reptiles | *Thamnophis elegans* | Tele | NCBI | GCF_009769535.1 |
| Reptiles | *Naja naja* | Nnaj | NCBI | GCA_009733165.1 |
| Reptiles | *Zootoca vivipara* | Zviv | NCBI | GCF_011800845.1 |
| Reptiles | *Parus humilis* | Phum | NCBI | GCF_000331425.1 |
| Reptiles | *Boa constrictor* | Bcon | figshare | https://doi.org/10.6084/m9.figshare.9793013.v2 |
| Reptiles | *Laudakia sacra* | Lsac | NGDC | GWHBKHB00000000 |
| Reptiles | *Gallus gallus* | Ggal | Ensembl | ftp.ensembl.org:/pub/release-102/fasta/gallus_gallus |
| Reptiles | *Sceloporus undulatus* | Sund | NCBI | GCF_019175285.1 |
| Fish | *Tetraodon nigroviridis* | Tnig | NCBI | GCA_051020865.1 |
| Fish | *Lates calcarifer* | Lcal | NCBI | GCF_001640805.2 |
| Fish | *Kryptolebias marmoratus* | Kmar | NCBI | GCF_001649575.2 |
| Fish | *Oryzias latipes* | Olat | NCBI | GCF_002234675.1 |
| Fish | *Gasterosteus aculeatus* | Gacu | NCBI | GCF_016920845.1 |
| Fish | *Boleophthalmus pectinirostris* | Bpec | NCBI | GCF_026225935.1 |
| Fish | *Danio rerio* | Drer | NCBI | GCF_049306965.1 |
| Fish | *Anabas testudineus* | Ates | NCBI | GCF_900324465.2 |
| Fish | *Takifugu rubripes* | Trub | NCBI | GCF_901000725.2 |
| Fish | *Sphaeramia orbicularis* | Sorb | NCBI | GCF_902148855.1 |
| Mammals | *Homo sapiens* | Hsap | NCBI | GCF_000001405.40 |
| Mammals | *Gorilla gorilla* | Ggor | NCBI | GCF_029281585.1 |
| Mammals | *Felis catus* | Fcat | NCBI | GCF_018350175.1 |
| Mammals | *Equus caballus* | Ecab | NCBI | GCF_002863925.1 |
| Mammals | *Elephas maximus* | Emax | NCBI | GCF_024166365.1 |
| Mammals | *Canis lupus familiaris* | Cfam | NCBI | GCF_000002285.5 |
| Mammals | *Sus scrofa* | Sscr | NCBI | GCF_000003025.6 |
| Mammals | *Bos taurus* | Btau | NCBI | GCF_002263795.2 |
| Mammals | *Mus musculus* | Mmus | NCBI | GCF_000001635.27 |
| Mammals | *Rattus norvegicus* | Rnor | NCBI | GCF_015227675.2 |
